# Effectiveness of short vs. long-distance sprint training on sprinting and agility performance in young soccer players

**DOI:** 10.5114/biolsport.2024.127384

**Published:** 2023-05-30

**Authors:** Ezequiel Rey, Samuel Carrera, Alexis Padrón-Cabo, Pablo B. Costa

**Affiliations:** 1 Faculty of Education and Sport Sciences, University of Vigo, Pontevedra, Spain; 2 Department of Physical Education and Sport Science, Faculty of Sports Sciences and Physical Education, Campus Bastiagueiro, University of A Coruña, Spain; 3 Human Performance Laboratory, Center for Sport Performance, Department of Kinesiology, California State University, Fullerton, CA, USA

**Keywords:** Association football, Physical fitness, Speed, Acceleration, Training load

## Abstract

The purpose of this study was to examine the effects of short sprint-distance training (SST) compared with long sprint-distance training (LST), matched for the total session training volume, on short-, medium- and long-distance sprint performance and agility in young soccer players. Eighteen U19 male players (age: 17.1 ± 0.7 years; height: 178.0 ± 6.3 cm, body mass: 69.4 ± 6.6 kg) were randomly assigned to SST (*n* = 9) or LST (*n* = 9) group. The intervention programs were performed 2 times a week over 6 weeks. Before and after training period, 5 m, 10 m, 20 m, 30 m and 40 m sprint, and agility were assessed. Within-group analysis showed significant improvements (*p* ≤ 0.001) in 5 m, 10 m, 20 m, 30 m and 40 m sprint from pretest to posttest in SST (9.2%, 6.6%, 5.3%, 2.9%, and 2.5%, respectively) and LST (10.5%, 8.5%, 6.5%, 5.1%, and 4.7%, respectively). Players in both SST and LST also showed significant enhancements in agility from pretest to posttest. In the between-groups analysis, there were no differences between the sprint training groups (SST vs. LST) in any variable (*p* > 0.05). In conclusion, the findings of this study indicate that both sprint training distances used seem to be effective to improve soccer-specific performance measures. However, due to the better percentage changes obtained by LST group in all fitness variables, this method could be considered as preferred method.

## INTRODUCTION

Soccer is characterized as an acyclical and intermittent high-intensity team sport in which short bouts of very intense activity are interspersed with lower intensity movements [1]. Time motion analyses have indicated that sprint account for ~4% and ~3% of the total distance covered during matches in adult and youth soccer players, respectively [2–4], and these actions are the most common and decisive movements during goal-scoring opportunities, that could determine the outcome of the game [5, 6]. Moreover, sprint ability can discriminate players from different standards of play [7]. In addition, professional soccer players have increased the peak sprint velocity, the total sprinting distance, and the number of sprints performed in match play over time [8]. Consequently, specific conditioning programs aimed to develop sprint should be considered paramount for physical performance in soccer [9, 10].

The importance of short-distance sprint in soccer, with the most common sprint during soccer matches varying between 2 to 4 s or 10 to 30 m, indicates that there is a large demand on acceleration speed [11]. However, sprint performance over medium-or longer-distances should also be developed as it is important for defensive and offensive success and injury prevention and differentiate between playing standards and age categories [12–15]. Thus, strength and conditioning coaches should use distance-specific training stimuli for their players to generate positive adaptations when attempting to enhance speed [16].

Sprint performance in soccer and other team sports can be improved through primary (e.g., sprint technique, sprinting), secondary (e.g., resisted or assisted sprinting), tertiary (e.g., non-specific methods including resistance training and plyometrics), or combined training methods [14, 17]. In this respect, primary training methods (e.g., sprint technique drills and un-resisted sprint) constitute the most used drills to develop sprint performance in elite football code athletes [9]. However, two recent systematic reviews and meta-analyses concluded that primary methods are insufficient to enhance performance in football code players [14, 17]. On the other hand, an early meta-analysis conducted on male youth team sport athletes, with 80% of the included studies focusing on soccer players, found that sprint training (which was the primary method) was an effective way to improve sprint performance. Furthermore, the study revealed that the effectiveness of sprint training increased progressively as the athletes matured. [18]. Thus, the training response to primary sprint training methods may be affected by mediator variables such as age or maturation status [14, 17].

To optimize training adaptations with sprint training in soccer, different principles of training (e.g., specificity, progression) and loading factors (e.g., intensity, recovery, frequency) may be followed and manipulated, respectively [4, 19]. Specifically, in soccer, several scientific protocols have been conducted to test the effect of manipulating different sprint training variables such as frequency (1 vs 2 days per week) [20], regime (linear vs. change-of-direction) [21], or intensity (maximal vs submaximal) [22, 23]. However, the distance covered per repetition in sprint training has not been explored in the literature. Despite the vast amount of scientific evidence on primary training methods in soccer, it is unclear what effects manipulating this variable under volume-equated conditions would have.

Considering the principle of specificity, short-sprint training should improve short-sprint performance, while longer sprints should improve medium- and/or long-sprint ability [4]. However, a scientific comparison between short and long sprint-training regimes remains unknown. Therefore, the aim of this study was to examine the effects of short sprint-distance training (SST) compared with long sprint-distance training (LST), matched for the total session training volume, on short-, medium- and long-distance sprint performance and agility in young soccer players. Considering the training specificity principle, it is hypothesized that SST would induce greater improvement in short sprint distances whereas LST would induce greater improvement in long sprint distances and agility.

## MATERIALS AND METHODS

### Design

This study used a two–group, randomized controlled trial design to compare the effects of different sprint training distances (SST vs. LSD). The intervention program of each group was added to the athletes’ daily training routine. The study was conducted over a 6-week competitive period (October–December) during the 2021–2022 season. During this period, the training regimen was designed to include a range of different drills and exercises, with a particular emphasis on technical and tactical development. These included technical drills, tactical drills, small-sided games, and game-based exercises. To compare the effects of sprint training, the following tests were selected: (a) 5 m sprint, (b) 10 m sprint, (c) 20 m sprint, (d) 30 m sprint, (e) 40 m sprint, and (f) T-test. To reduce the influence of confounding variables, all subjects were instructed to maintain their usual lifestyle and normal dietary intake before and during the course of the study.

### Subjects

A priori power analysis [24] (G*Power, version 3.1.9.7, Universität Kiel, Düsseldorf, Germany) with an assumed type I error of 0.05 and a type II error rate of 0.20 (80% statistical power) was conducted for sprint performance. It revealed that eight subjects per group would be sufficient to observe medium group × time interaction effects. Eighteen U19 male soccer players were recruited for the current study. Exclusion criteria were injuries resulting in the loss of one or more soccer matches/training sessions in the three months prior to study initiation. Only outfield players were included (i.e., the goal-keepers were excluded). The participants in systematic soccer training had a mean experience of 9.08 ± 3.27 years. The players regularly performed 4–5 weekly soccer sessions with their team on average exercising 8.1 ± 2.2 h · wk^−1^ in their normal training cycle. Likewise, the team usually competed in one official match per week. Players were randomly assigned by an investigator not directly involved in testing or the training intervention into 1 of 2 groups, SST (n = 9; age: 17.1 ± 0.7 years; height: 177.4 ± 5.9 cm, body mass: 71.5 ± 7.11 kg) or LST (n = 9 age: 17.1 ± 0.8 years; height: 178.6 ± 7.1 cm, body mass: 66.5 ± 5.2 kg). The intervention program was added to the usual training routines. In all other respects, all subjects completed identical training activities. Only players who participated in at least 80% of all training sessions were included in the statistical analysis. Written informed consent indicating their voluntary participation was obtained from participants and legal representatives after explanation of the experimental protocol and its potential benefits and risks. The research protocol was approved by the Local Ethics Committee (University of Vigo; 20–0320), in accordance with the Code of Ethics of the World Medical Association (Declaration of Helsinki).

### Training Programs

After pretesting, subjects began one of the six-week sprint training protocols presented in Table 1 in addition to the usual soccer training. The intervention program was performed 2 times a week (total of 12 sessions), on non-consecutive days (4 days and 2 days before match). This schedule remained consistent throughout the entire 6-week period. The training sessions were conducted on an artificial pitch turf, which was the same surface used for the testing sessions. Both groups completed the same amount of total distance per session (Table 1). The only difference between the 2 interventions was that the SST group performed all the maximal straight-line sprints in a distance of 20 m and the LST group in 40 m. The players were instructed to provide maximal effort in each training session. Before each session, participants completed a standardized warm-up (same as pre- and post-testing), as prescribed by a certified strength and conditioning specialist. A certified strength and conditioning specialist supervised all training sessions to ensure that all warm-up activities and sprints were completed with correct technique and with maximum effort. Foster’s 0–10 scale was recorded to quantify the intensity of the training sessions using rating of perceived exertion (RPE) [25]. All participants were familiarized with the use of this RPE scale, as they had used it throughout the season in their teams’ training sessions.

**TABLE 1:** Summary of training load progression.

Week	Group	Distance (m)	Recovery (s)	Repetition	Distance per session (m)	Distance per week (m)
1	SST	20	60	12	240	480
	LST	40	120	6	240	480
2	SST	20	60	12	240	480
	LST	40	120	6	240	480
3	SST	20	60	14	280	560
	LST	40	120	7	280	560
4	SST	20	60	14	280	560
	LST	40	120	7	280	560
5	SST	20	60	16	320	640
	LST	40	120	8	320	640
6	SST	20	60	16	320	640
	LST	40	120	8	320	640

SST = short-sprint training; LST = long-sprint training

### Procedures

During testing sessions, the players were required to wear the same athletic equipment and measurements were conducted at the same time of the day to minimize the effect of diurnal variations on the selected parameters during two experimental sessions. All data collection and test sessions were performed on the same pitch. Each player was instructed and verbally encouraged to make a maximal effort during all tests. All tests were performed after 72 hrs of rest and at the same venue under identical conditions and supervised by the same investigators. The players complied with the following pre-test guidelines: (a) to not consume any caffeinated beverages or supplements 48 hrs prior to testing; (b) to not consume food at least 2 hours prior to testing. Before testing, all participants performed 10 min of standardized warm-up involving 2 min of light dynamic stretching (10 repetitions for hamstrings, quadriceps, and calf muscles) and 5 min of jogging, followed by short distance accelerations (3 submaximal sprints, progressing to 90% of their maximal velocity for the shuttle distance [30 + 30 m]). This routine was supervised by the team’s coach before the tests. During testing sessions, players performed the following tests:

*5 m, 10 m, 20 m, 30 m, and 40 m sprint tests.* Sprint time was measured using a dual infrared reflex photoelectric cell system (Witty, Microgate, Bolzano, Italy). To capture data, five pairs of wireless photoelectric cells were mounted on tripods at a height of 0.9 m and spaced at intervals of 0, 5, 10, 20, 30, and 40 m. All players began with a standing start, with the front foot positioned 0.5 m from the first timing gate. They were instructed to perform all the sprints with a maximal effort. To ensure reliable and consistent data, each participant was given three attempts, with a 3-minute recovery period allowed between each trial.

*T-test.* Photoelectric cells (Witty, Microgate, Bolzano, Italy), placed on the starting line, were used to measure the soccer players’ performance and to increase test reliability. A T-test was administered using the protocol outlined by Munro and Herrington [26]. Participants performed three trials, and the fastest time was used as the T-test score. When ready, players sprinted forward 9.14 m to touch the first cone. They then side-shuffled 4.57 m to the left and touched the second cone. Next, they side-shuffled 9.14 m to the right and touched a third cone, and then 4.57 m to the left, back to the point where the first cone was, touching it again. Finally, participants back-pedaled 9.14 m, passing through the finish line.

### Statistical Analysis

All variables were normally distributed (Shapiro-Wilk test). Data are presented as means and standard deviation (SD). All statistical analyses were conducted using the statistical package SPSS for Macintosh (version 25.0, Chicago, IL, USA). A 2 (group: SST and LST) × 2 (time: pre, post) mixed factorial analysis of variance (ANOVA) was calculated for each parameter. Additionally, Cohen’s *d* was computed for comparing effect sizes (ES). ES were classified as trivial (*d <* 0.2), small (0.2 ≤ *d <* 0.5), moderate (0.5 ≤ *d <* 0.8), and large (≥ 0.8) [27]. Moreover, pre- to-post change percentage was calculated for corresponding variation. Relative and absolute reliability of the variables analyzed in this study were assessed using the intraclass correlation coefficient (ICC) and the coefficient of variation (CV), respectively. Significance was established at the P ≤ 0.05 level.

## RESULTS

Reliability results are shown on Table 2. The relative reliability as depicted by ICC was very high for all the tests, exceeding 0.80 (ranging from 0.86 to 0.98). The absolute reliability also showed very high levels for all the test with CV ranged from 2.93 to 0.49%. RPE scores collected at each training session during the whole training period were not different between the two groups (p > 0.05; 2.5 and 2.6 for SST and LST, respectively).

**TABLE 2:** Relative and absolute reliability measures for the assessed variables.

Variables	ICC	CV (%)
5 m	0.90	1.8
10 m	0.92	1.1
20 m	0.94	0.8
30 m	0.98	0.5
40 m	0.97	0.5
T-test	0.86	2.9

ICC = intraclass correlation coefficient; CV = coefficient of variation.

Mean values and SD, percentage changes from pre- to post-training for 5 m, 10 m, 20 m, 30 m, 40 m sprint tests, and T-test performance indices are reported on Table 3.

**TABLE 3:** Changes in physical fitness after six weeks of sprint-training in youth soccer players.

Variables	SST				LST				ANONA		
	Pre	Post	Δ (%)	ES	Pre	Post	Δ (%)	ES	Time	Group	Time × group
**5 m (s)**	1.08 ± 0.04	0.98 ± 0.06	-9.2	1.96	1.09 ± 0.02	0.98 ± 0.03	-10.5	4.31	< 0.001	0.657	0.437
**10 m (s)**	1.82 ± 0.07	1.70 ± 0.08	-6.6	1.59	1.85 ± 0.05	1.70 ± 0.03	-8.5	3.63	< 0.001	0.536	0.055
**20 m (s)**	3.14 ± 0.12	2.98 ± 0.14	-5.3	1.22	3.19 ± 0.07	2.98 ± 0.08	-6.5	2.79	< 0.001	0.656	0.129
**30 m (s)**	4.42 ± 0.23	4.28 ± 0.22	-2.9	0.62	4.45 ± 0.13	4.23 ± 0.10	-5.1	1.89	< 0.001	0.933	0.216
**40 m (s)**	5.66 ± 0.33	5.51 ± 0.28	-2.5	0.49	5.71±0.18	5.43 ±0.11	-4.7	1.87	0.001	0.910	0.217
**T-test (s)**	9.64 ± 0.49	9.17 ± 0.27	-4.7	1.18	9.53 ± 0.33	9.00 ± 0.55	-5.5	1.16	< 0.001	0.469	0.731

SST = short-sprint training; LST = long-sprint training; ES = effect size.

There were no significant group time × group interactions observed in any of the sprint and agility tests (p > 0.05). A significant time effect was found in the 5 m, 10 m, 20 m, 30 m, and 40 m sprint tests for SST and LST. The statistical analysis also revealed main effect for time in the T-test for SST and LST.

**FIG. 1:**
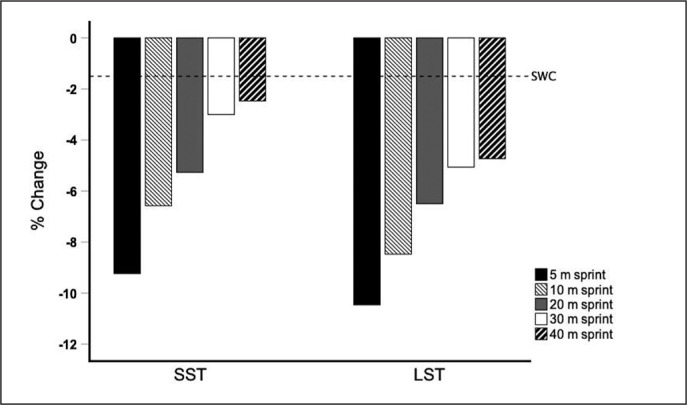
Percentage change in sprint performance in response to short (SST) and long-sprint training (LST). Horizontal line represents the smallest worthwhile change (SWC) for team sports athletes.

The percentage change for SST and LST groups in the sprint tests is shown in Table 3 and Figure 1.

## DISCUSSION

The aim of this study was to analyze the effect of a sprint training protocol with short and long distances in youth soccer players during the in-season period. To our knowledge, this is the first sprint-training study that has been conducted in soccer players comparing the effects of different sprint training distances on physical fitness. Based on the analyses, the main findings of this study were that: (a) both sprint training interventions were equally effective in developing 5 m, 10 m, 20 m, 30 m, and 40 m sprint performance; (b) both SST and LST induced significant changes in T-test performance.

Sprinting speed is one of the most essential fitness components for playing soccer [5, 6]. Moreover, sprint ability can discriminate youth players from different standards of play [7]. Therefore, training interventions aimed at improving sprinting speed may be a priority for youth soccer coaches. In contrast to the main research hypothesis, both the SST and LST training programs induced similar significant and positive changes in all sprint distances, without significant differences between both sprint training programs. The rationale for this hypothesis was based on the training specificity principle as short sprints would be more effective in developing acceleration in short distances (e.g., 5 m, 10 m, and 20 m sprint) than the long repetitions, despite the matched total distance of both training programs.

Haugen and Buchheit [28] stated that the smallest worthwhile change (SWC) for team sport players is ~1.5% for 5 m sprints and ~1% for 10 to 40 m sprints. Since the performance changes observed in the present study were clearly greater (ranged between 2.5% to 10.5%) than the measurement noise observed (ranged between 0.5 to 1.8% CV for sprint time) and the SWC described in the scientific literature for team sport players [28, 29], the usefulness of the SST and LST protocols performed was reasonably high (Figure 1).

This is the first study that compared the effects of different sprint training distances on short, medium, and long sprint performance, therefore, direct comparisons with other studies are not possible. Nevertheless, the main results of the present study are consistent with previous investigations that examined the effects of primary sprint training method on sprint performance in soccer players with similar age group cohort. For example, Pavillon et al. [21] compared the effect of two different sprint training regimes (i.e., linear sprints vs. change-of-direction sprints) on short-distance sprint performance in youth soccer players over 30 weeks. The results showed significant improvements in 5 m and 10 m sprint performance. Likewise, Marzouki et al. [20] reported one or two sprint training sessions per week of equal volume produce similar improvements in 10 m, 20 m, and 30 m sprint performance in youth soccer players. In addition, the present study observed performance changes across different sprint distances in both the SST and LST groups, ranging between 2.5% to 9.2% and 4.7% to 10.5%, respectively. These findings are consistent with previous studies in professional, who experienced similar improvements in sprint performance after a 6-week training program involving a combination of resisted (2.7% to 6.9%) and unresisted (2.1% to 8.4%) methods such as squat jumps, linear sprints, and change-of-direction drills [30]. Additionally, similar changes were observed in youth soccer players who completed a 5-week high-in-tensity interval training (5.0% to 7.3%) and small-sided games (5.9% to 7.9%) programs [31].

The present findings are in line with the pattern of sprint trainability described by Moran et al. [18] in their meta-analysis regarding the effects of sprint training on sprinting performance across peak height velocity groups (PHV) in young male athletes. As an outcome of this article, the authors stated that sprint training becomes progressively more effective with increasing maturation showing the post-PHV group the greatest trainability effects, which corresponds with the participants of the present study in terms of chronological age (16–18 years). Thus, large effects observed in SST and LST could be explained by the greater muscular size, hormonal activity and development, greater muscular size, increased limb length, changes to musculotendinous tissue, enhanced neural and motor development and better movement quality and coordination [18, 32].

Agility is considered an important quality required by team sports players [33]. According to previous literature, training programs designed to improve agility should be specific and independent from sprint training programs [34]. However, in the present study SST and LST groups induced improvements of 4.7 and 5.5% in the T-test, respectively, similar to the effect observed in sprint performance. However, no significant difference was observed between the two experimental groups, suggesting that agility improvements are not dependent on sprint training distance when players perform the same training volume. These findings are in accordance with the results of Marzouki et al. [20], who reported a significant reduction of 4.2 and 2.4% in T-test performance after 10-week training including on or two sessions a week, respectively. Furthermore, the current study’s results are consistent with those of Bianchi et al. [35] who demonstrated a significant decrease in the 505 change-of-direction test time after implementing a 6-week combined training method involving both plyometrics and sprinting drills. Several factors could explain the agility improvements observed in this study after training period. However, the most plausible factor could be related to in-crements in lower limb strength [21].

The results of the present study suggest that sprint training programs with short or long distances were both useful for improving sprint performances over distances between 5 and 40 m. Indeed, present results demonstrated the prescription of SST or LST during in-season period contributed to improving agility performance among youth soccer players. These results reinforce previous evidence indicating usual sprint training modality is an approach to be recommended to increase sprint performance in male youth athletes [18].

The interpretation and broader implications of the current findings must be understood within the limits of the specific data collection undertaken. Although the study had many unique aspects, there are some limitations that should be considered. First, even though the number of participants in this study was similar to other sprint training studies in youth soccer players. Another limitation to be considered in this study is the duration of the training intervention, which was only six weeks. Third, the absence of a control group without participating in any of the experimental protocols limits conclusions from this study. Future studies considering a larger sample size, longer training periods, and using control group may provide more conclusive results.

## CONCLUSIONS

This study showed that six weeks of short- and long-distance sprint training, matched for the total session training volume, seem to represent a time-efficient stimulus for a simultaneous improvement of short, medium, and long sprint performance, as well as agility during in-season period in youth soccer players. However, although there were no statistically significant differences between the two training programs, LST group showed better percentage changes in all fitness variables evaluated. Thus, from a practical perspective, because even small changes can be the difference between winning and losing decisive 1-on-1 duels or create goal-scoring opportunities in soccer by having body or shoulder in front of the opposing player, LST seems to be a preferred training method for these variables. These training-specific adaptations offer coaches and strength and conditioning professionals the possibility to individualize training content specific to the athletic qualities in soccer.
